# AllergoOncology in Review: Harnessing Allergy in the Field of Oncology to Improve Patient Outcomes

**DOI:** 10.1111/cea.70234

**Published:** 2026-02-11

**Authors:** Jakub Zydron, Anishaa Balaji, Jitesh Chauhan, Jack Alder, Xinyi Chen, Anna M. F. Wiegman, Aurelie Poli, Joanna Jacków‐Malinowska, Jack Cheeseman, Daniel I. R. Spencer, James McDonnell, James Spicer, Alexandra J. McCraw, Sophia N. Karagiannis

**Affiliations:** ^1^ St. John's Institute of Dermatology, School of Basic and Medical Biosciences & KHP Centre for Translational Medicine King's College London Guy’s Hospital London UK; ^2^ School of Cancer and Pharmaceutical Sciences King's College London, Guy's Hospital London UK; ^3^ Randall Centre for Cell and Molecular Biophysics, School of Basic and Medical Biosciences King's College London London UK; ^4^ Neuro‐Immunology Group, Department of Cancer Research Luxembourg Institute of Health Luxembourg Luxembourg; ^5^ Ludger Ltd., Culham Campus Abingdon Oxfordshire UK; ^6^ Breast Cancer Now Research Unit, School of Cancer and Pharmaceutical Sciences, Guy's Cancer Centre King's College London London UK

**Keywords:** AllergoOncology, allergy, cancer, IgE, immune surveillance, immunotherapy

## Abstract

The AllergoOncology field brings together the study of allergic and cancer immune responses, having evolved from early epidemiological studies that reported inverse associations between allergies, IgE and cancer risk. Insights from studying allergic inflammation are revealing previously unappreciated immune mechanisms that confer protective effects against cancer, and evasion pathways that facilitate tumour progression. AllergoOncology sheds light on cancers with poor prognosis, including glioma, where evidence has pointed to allergic triggers that may influence glioma biology through rewiring immune surveillance. Allergic signals point to new biomarkers that may identify groups at higher risk of developing cancer, aid patient stratification and help monitor treatment and clinical outcomes. Allergic mediators such as histamine and IgE levels are emerging biomarkers that can inform cancer risk and lead to clinical interventions that improve outcomes. Emerging cancer immunotherapies, such as tumour antigen‐specific IgEs, an evolving therapy class, are and will continue to be inspired by understanding allergic immune response mechanisms. Assays, including the Basophil Activation Test developed for monitoring and managing allergic reactions, are translated to the oncology clinic to evaluate hypersensitivity to anti‐cancer therapeutics. Allergy research brings fundamental benefits for oncology through understanding and harnessing allergic and cancer‐associated mechanisms in AllergoOncology for patient benefit.

## Key Points

- AllergoOncology explores the intricate links between allergic responses and cancer immune surveillance, treatment and outcomes.
- Insights from allergy enhance how we understand cancer immunity, manage drug reactions and develop therapeutics.
- Immunotherapies and biomarkers derived from shared pathways in allergies and cancer will advance precision medicine.

**Abbreviations**
AAI: Allergic airway inflammation
ADCC: Antibody‐dependent cell‐mediated cytotoxicity
ADCP: Antibody‐dependent cell‐mediated phagocytosis
BAT: Basophil Activation Test
BC: Basophil count
Breg: Regulatory B cell
CNS: Central nervous system
CRC: Colorectal cancer
CSPG4: Chondroitin sulfate proteoglycan 4
CVD: Cardiovascular disorder
DC: Dendritic cell
DCreg: Regulatory dendritic cell
DHR: Drug hypersensitivity reactions
DPP4: Dipeptidyl peptidase IV
EAACI: European Academy of Allergy & Clinical Immunology
EC: Eosinophil count
EGFR: Epidermal growth factor receptor 1
FRα: Folate receptor alpha
GBM: Glioblastoma
GI: Gastrointestinal
GMP: Good Manufacturing Practice
HER2: Human epidermal growth factor receptor 2
ICI: Immune checkpoint inhibitor
IDH: Isocitrate dehydrogenase
IDH‐WT: IDH‐wild‐type
IEI: Inborn errors of immunity
IgEG: Hybrid IgE‐IgG1
IMP: Investigational medicinal product
IrAE: Immune‐related adverse events
MC: Mast cells
NET: Neutrophil extracellular trap
NSCLC: Non–small‐cell lung cancer
PAMP: Pathogen‐associated molecular pattern
PSA: Prostate‐specific antigen
rIgE: Recombinant IgE
TAM: Tumour‐associated macrophages
Th1: T‐Helper 1
Th2: T‐Helper 2
TLR: Toll‐like receptor
TME: Tumour microenvironment
Treg: Regulatory T cell

## 1 Introduction

The emerging field of AllergoOncology explores the intriguing relationship between Th2 immunity, allergic responses and cancer. Immune cells, inflammatory mediators, signalling cascades and antibodies such as IgE and IgG4, known to participate in allergic diseases, may form part of tumour immune surveillance and contribute to cancer prevention, immune responses and clinical outcomes.

The origins of AllergoOncology can be traced back to early reports of associations between allergic conditions and the risk of different cancers [1, 2, 3, 4]. While some research suggested that individuals with allergies were less likely to develop certain cancers, other studies indicated that certain allergic diseases, such as asthma carried a heightened risk for specific malignancies such as lung cancer [5, 6]. These intriguing observations, alongside expanding research activity in the area of cancer immunology and immunotherapy, heightened interest in exploring the intricate interplay between aspects of immune responses and their links to tumour biology [7, 8, 9, 10, 11, 12, 13, 14, 15, 16, 17]. Some studies highlighted parallels between allergic inflammation and anti‐ or pro‐tumour immune surveillance, precipitating AllergoOncology as an expanding multidisciplinary field.

Current research efforts include identifying prognostic links between allergy/atopy and cancer risk or cancer outcomes, uncovering fundamental immune mechanisms that may help better explain both allergic and malignant conditions and identifying aspects of allergic inflammation which could be harnessed for the identification of biomarkers as well as precision medicine approaches [9]. The latter encompasses therapeutics development presently exemplified by the design, engineering, evaluation and translation of IgE antibodies directed to tumour‐associated antigens for cancer therapy [1, 9]. Several position papers have been published within the field (Table 1).

**TABLE 1:** Summary of position papers focusing on different areas of AllergoOncology and future research directions in the field arising from these.

Paper title and publication date	Areas of focus	Key points	Future research directions
Basophil Activation Test: Bridging allergy and oncology for diagnostic, therapeutic and prognostic applications in AllergoOncology: An EAACI position paper Pascal et al. (2025) [160]	The role of BAT; an ex vivo functional assay in allergy to assess IgE‐driven hypersensitivity to food allergens, medications, venoms, and to track tolerance development Diagnostic, therapeutic, and prognostic applications of BAT within the field of AllergoOncology	Establishing standardised workflows including marker selection (such as CD63 and CD203c), stimulation protocols, and data analysis criteria to achieve reproducible results, refine patient stratification, and monitor safety during the development of IgE‐based cancer treatments Beyond its diagnostic role, the BAT delivers prognostic and therapeutic insight: serial measurements of basophil activation can predict clinical outcomes and guide tolerance‐induction strategies in allergy‐focused oncology trials	Use in predicting and monitoring of IgE‐mediated hypersensitivity to chemotherapeutics and biologics to distinguish true type I hypersensitivity from other reactions Widespread adoption of the BAT in AllergoOncology to enhance personalised care through better diagnosis, biomarker‐driven patient stratification, and real‐time therapy monitoring
Granulocytes and mast cells in AllergoOncology‐Bridging allergy to cancer Pascal et al. (2024) [52]	The role of granulocytes and mast cells in cancer Interactions between allergy and cancer Allergic inflammation and the tumour microenvironment	Granulocytes and mast cells have a complex, context‐dependant, role within cancer. In some settings, they can be pro‐tumour (promoting growth and metastasis) or anti‐tumour (cancer cell cytotoxicity and preventing metastasis) Mast cells and granulocytes may impact the efficacy of cancer therapies, including immunotherapies	Clarify the precise mechanisms by which granulocytes and mast cells influence cancer biology Investigate the therapeutic potential of targeting mast cells and granulocytes in cancer therapies To validate biomarkers for prognostic and therapeutic outcomes and associated toxicities
Biomarkers and refined classification for research in the allergy and glioma nexus Turner et al. (2024) [32]	The relationship between allergic diseases and glioma and the protective role of allergies in glioma development Can specific allergy biomarkers help identify individuals at risk of glioma	Key biomarkers are needed to identify patients who are at a higher or lower risk of developing glioma Allergic inflammation has multiple roles within glioma development. Allergy response may decrease the risk of glioma, but chronic inflammation may create a pro‐tumour environment	Identifying specific biomarkers linked to allergic disease and Glioma progression Refine glioma classification to incorporate immune‐associated features to inform treatment decisions Further explore clinically relevant biomarkers and explore more glioma sub‐classifications
Danger signals in allergology and oncology Bergmann et al. (2022) [7]	How DAMPs and PAMPs activate the innate immune system causing inflammation How mast cells and granulocytes respond to danger signals How DAMPs and PAMPs can shape the Tumour microenvironment	Danger signals are context dependant, can exhibit immune surveillance and anti‐tumour responses or pro‐tumour activity Allergy and cancer both involve TLRs and inflammasome signalling, but downstream responses differ due to differences in immune regulation and antigen type Danger signal modulators may have potential application in allergy treatment and cancer immunotherapy	Further research is required to identify the specific DAMPs and PAMPs that drive inflammation in allergy and cancer settings Develop targeted therapies to modulate danger signal pathways to dampen allergic inflammation or enhance anti‐tumour activity
Ultra‐low IgE, a potential novel biomarker in cancer Ferastraoaru et al. (2020) [24]	The role of IgE in immune surveillance and anti‐tumour responses ‘Ultra‐low’ IgE being a risk factor of increased malignancy susceptibility Low IgE levels and their ability to be used as a predictive marker for increased cancer susceptibility	Data showed that individuals with higher IgE levels experienced a lower risk of developing certain cancers (glioma, CLL and MM) Individuals with low/absent IgE levels may be at a higher risk for malignancies	Validate the association between ultra‐low IgE and cancer across different demographics and differing cancer types To find out if the prevalence of IgE deficiency is age dependant To make total serum IgE testing a routine measurement for risk assessment protocols
Microbiota in allergy and cancer Untersmayr et al. (2019) [16]	The role the microbiota plays in shaping both innate and adaptive immune responses The impact of lifestyle on microbiota composition and how these influences can affect cell communication How imbalances in microbiota composition are correlative to immune‐related disease and cancer	A balanced microbiota is associated with protective health effects and reducing the risk of immune‐related diseases There is an association between dysbiosis with allergy and cancer with microbiota imbalances Microbiota composition can influence patient treatment responses in both allergy and cancer	Develop interventions aimed at restoring the microbiota composition in patients to improve treatment outcomes Incorporate microbiota profiling into personalised treatment plans to predict and enhance patient treatment response
Opposite outcomes of immune tolerance in allergy and cancer Jensen‐Jarolim et al. (2018) [10]	Contrasting immune tolerance mechanisms in both allergy and cancer Explores the roles of how Tregs and Bregs maintain immune homeostasis in allergy and suppressing anti‐tumour immunity The impact of cytokines (IL‐10 and TGF‐b) and chemokines (CCL1 and CCL5) in allergy and cancer progression	Immune tolerance mechanisms can be co‐opted by tumours to supress immune responses and promote cancer progression Tregs and Bregs may contribute to an immunosuppressive TME, impacting anti‐tumour immunity Cytokines can promote tumour immune evasion whilst chemokines recruit immunosuppressive cells to the tumour site	Further research into the mechanisms at play between immune tolerance and disease progression and patient outcomes in both allergy and cancer therapy
The impact of allergy in oncology Jensen‐Jarolim et al. (2017) [9]	How Th2 immunity and IgE‐mediated responses influence tumour surveillance and progression The role of IgE and associated effector cells in cancer cell recognition and elimination	IgE has displayed a dual role in allergic reactions and may also contribute to anti‐tumour immunity through ADCC Preclinical studies have shown that IgE antibodies targeting tumours can inhibit tumour growth RDD protocols have enabled patients with hypersensitivity to chemotherapeutic agents to continue treatment safely	Identify key markers that are predictive of a patient's response to an IgE‐mediated therapy Uncover the mechanistic pathways through which Th2 immunity and IgE contribute to tumour surveillance Incorporation of allergy assessment in oncology protocols to personalise treatment plans to manage hypersensitivity reactions
The role of IgE‐mediated allergy in cancer Jensen‐Jarolim et al. (2008) [1]	The role of IgE in tumour immunity and in tumour cell detection and elimination Looking at how IgE‐armed effector cells can mediate anti‐tumour activities Looking at the role of IgE‐binding effector cells in mediating anti‐tumour activities Cross‐presentation mechanisms of IgE facilitating the uptake and presentation of tumour cells to DCs	IgE antibodies may contribute to the body's natural defences against rumours by sensitising effector cells to recognise and eliminate cancer cells Recombinant IgE antibodies targeting TAAs have demonstrated efficacy in preclinical models IgE has shown to display effector cell activation in which it activates and stimulates the release of cytotoxic	Advancing recombinant IgE antibodies in clinical trials to assess safety and efficacy in cancer patients To begin to identify biomarkers that are predictors of patient responses to IgE‐based therapies Explore the integration of IgE‐based therapies with pre‐existing cancer treatments to enhance their overall efficiency

Abbreviations: ADCC, antibody‐dependant cell‐mediated cytotoxicity; BAT, basophil activation test; Bregs, regulatory B cells; CCL, Chronic lymphocytic leukaemia; CCL1, chemokine ligand 1; CCL5, chemokine ligand 5; DAMPs, Damage‐associated molecular patterns; DC, dendritic cells; IgE, immunoglobulin E; IL‐10, interleukin 10; MM, multiple myeloma; PAMPs, pathogen‐associated molecular patterns; RDD, rapid drug desensitisation; TAAs, tumour‐associated antigens; TGF‐b, transforming growth factor beta; Th2 immunity, type 2 immunity; TLR, toll‐like receptor; TME, tumour microenvironment; Tregs, regulatory T cells.

In this review, we summarise key areas of the rapidly advancing AllergoOncology field including updates on key topics previously discussed in different position papers and highlight potential future directions and benefits within personalised medicine and cutting‐edge immunotherapies arising from insights in AllergoOncology [1, 18, 19, 20, 21]. We point to epidemiological links between allergy and cancer underlying the search for putative biomarkers, including neuro‐oncological links that provide functional insight into mechanisms of immune modulation in challenging cancers such as glioma, to highlight the most prominently reported epidemiological link between allergy and cancer. We discuss immune cells with known roles in allergy that also participate in tumour inflammation and the use of established assays in the field of allergy in oncology applications. Finally, the development of IgE‐based anti‐tumour therapeutics, including the first‐in‐class therapeutic IgE antibody candidate for cancer, MOv18‐IgE, may complement existing antibodies currently only utilising the IgG class. Herein, we summarise developments in these research areas, highlight potential limitations and discuss the future and new directions in AllergoOncology.

## 2 Epidemiological Links Between Allergy and Cancer

**FIGURE 1:**
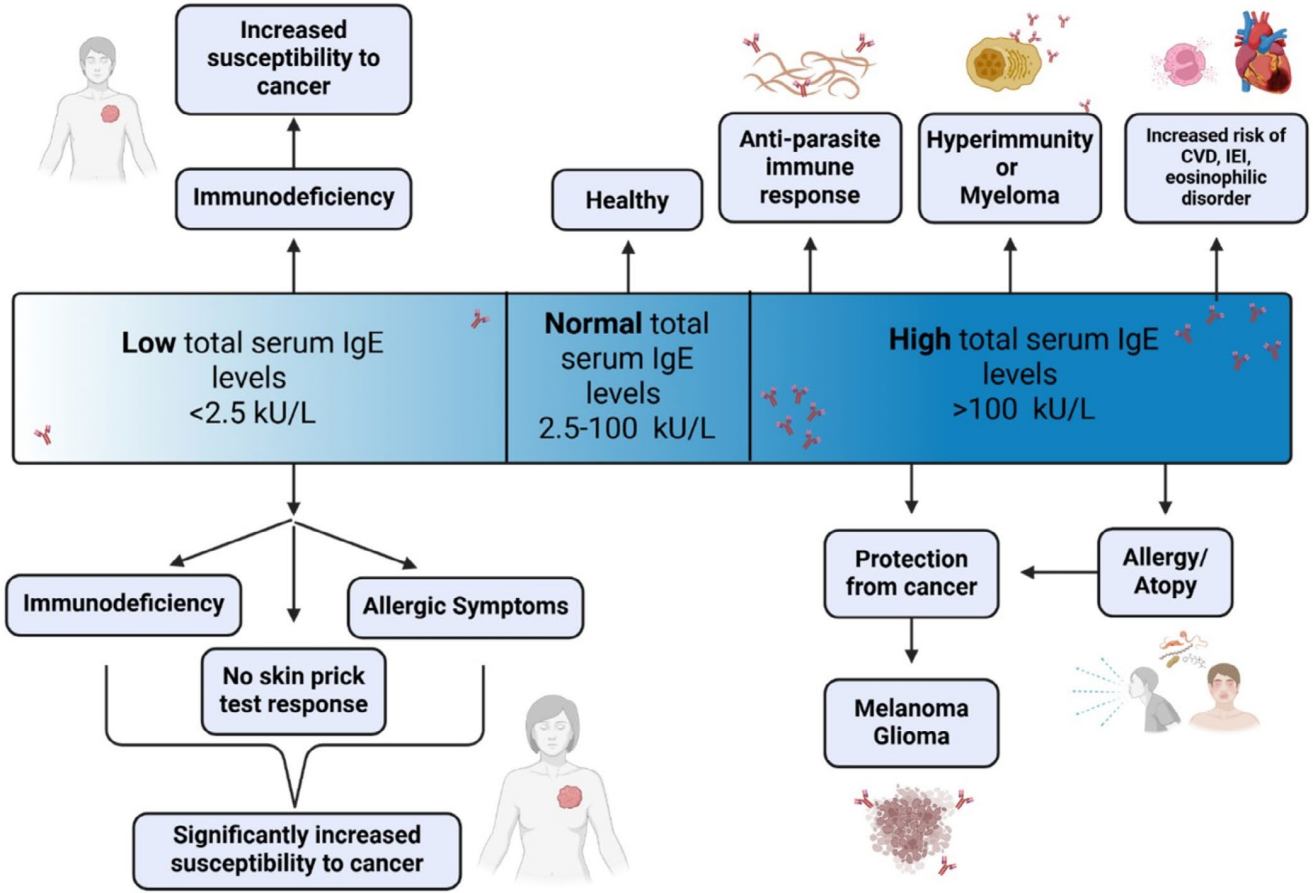
Implications of total serum IgE levels in health and different diseases [3, 13, 15, 22, 23, 24, 25, 28, 32, 157, 158]. Levels of serum IgE have been reported to be associated with different diseases or homeostatic states (boxes and images above the IgE serum level bar). Levels of serum IgE are also reported to associate with significantly increased susceptibility to cancer (boxes and images below the IgE serum level bar). Low total serum IgE levels indicate immunodeficiency and higher rates of malignancy (boxes and images above the IgE serum level bar). Ultra‐low IgE levels as part of an immunodeficiency diagnosis in the absence of other allergic conditions (e.g., rhinitis) show stronger associations with malignancy risk, suggesting that lack of IgE‐driven immune activity may denote impaired tumour immunosurveillance. Patients with ultra‐low IgE who have negative skin prick tests, reflecting a non‐atopic immune profile, may correlate with reduced immune vigilance against tumour cells and increased cancer susceptibility (boxes and image below the IgE serum level bar). Normal total serum IgE levels may be part of immune surveillance and homeostasis (healthy). High total serum IgE levels are associated with atopic/allergic and parasitic conditions, hyperimmunity, myeloma and increased risk of cardiovascular disorders (CVD), inborn errors of immunity (IEI) and eosinophilic disorder (boxes and images above serum level bar). Higher IgE levels and allergy may be associated with lower risk of certain cancers (e.g., melanoma or glioma; boxes and images below the IgE serum level bar). Created with BioRender.com.

Epidemiological associations have been reported both between allergy/atopy symptoms and diagnosis and cancer risk [3, 13, 22, 23] and also between IgE levels and cancer risk [15, 24, 25] (Figure 1). There is a consensus for an inverse association between allergies and cancer development when including all cancers [5, 6, 26, 27]. However, specific associations are also observed when stratifying by cancer type. For example, allergy/atopy is negatively associated with the risk of developing basal cell carcinoma or malignant melanoma [28], and hay fever is negatively associated with pancreatic cancer risk [29]. Links with breast and prostate cancers, however, are less clear [26, 27]. Asthma in the absence of other allergies appears to be associated with increased lung cancer risk [26, 30], although evidence may be inconsistent across different studies [31]. Glioma (discussed below) appears inversely associated with allergy/atopy, but several other factors may influence risk, including ethnicity, antihistamine use and the environment [32].

Overall, the relationship between allergy/atopy and cancer risk appears highly contextual and may depend on multiple factors including the type of allergic/atopic disease studied, the anatomical site of the primary cancer linked to the specific tissue environment and immune mediators and immune cells present in different tissues and under certain inflammatory conditions [2, 13]. Treatments may also potentially contribute to these complex interactions: for instance, the use of antihistamine may abrogate any protective effects of allergy against some types of cancers [33], but following cancer onset, histamine may support Th2 inflammation and instead negatively impact therapeutic response to immune checkpoint inhibitor (ICI) antibodies such as nivolumab (anti‐PD‐1) [34] and ipilimumab (anti‐CTLA‐4) [35, 36]. Several hypotheses have been put forward to explain the relationship between allergy/atopy and cancer (Table 2). The most recent of these is the combinatorial hypothesis that the complex interplay of symptomology, symptom management, Th2 immune skewing, and factors such as IgE levels must be considered as a whole to provide insight into an individual's cancer risk [13].

**TABLE 2:** Proposed hypotheses to explain the relationships between allergy and/or atopic disease, IgE with cancer.

Hypothesis	Summary and examples	Impact on cancer risk	References
Chronic inflammation	Allergic symptoms drive tissue inflammation, leading to mutation of tumour suppressors and other genes involved in cell growth regulation, correspondingly increasing cancer risk at sites of chronic inflammation	Increased	[2]
	Atopic dermatitis linked to increased skin cancer risk		[161]
	Asthma linked to increased lung cancer risk		[26, 30]
Immunosurveillance	Allergy reflects immune hyper‐responsiveness and enhanced natural immunosurveillance as manifested by elevated serum IgE. Tumour immunosurveillance is heighted as a result	Decreased	[2]
	Low IgE levels are associated with heightened cancer risk		[24, 25]
	General decreased risk of cancer associated with allergy and/or atopy		[2, 3, 5, 26]
Prophylaxis	Physical symptoms of allergy—e.g., coughing, sneezing—can expel potential carcinogens, decreasing cancer risk	Decreased	[2, 162]
	Decreased risk of upper gastrointestinal cancers associated with allergy and respiratory atopic disease		[163]
	Antihistamine use may increase cancer risk for specific cancers		[41]
Th2 immune skewing	Inappropriate skewing towards T‐helper‐2 (Th2)‐based responses creates a more tumour‐permissive immune environment. Tissues affected by atopic disease are more prone to cancer development	Increased	[2]
Combinatorial	Individual factors of specific local and systemic allergic disease, general immune responsiveness including IgE levels, chronic inflammation and symptomology as well as symptom management must be considered in the context of cancer risk. Cancer risk may, for example, be heighted at sites of allergic inflammation where chronic inflammation could lead to cell exhaustion and regulatory cell accumulation in the inflamed tissue; but decreased at distal sites due to stimulation of classical Th2 responses. Furthermore, the use of medications such as antihistamines and anti‐inflammatory medications may modulate cancer risk	Variable depending on context	[13]
	Asthma appears protective against different cancers, but it is associated with increased risk of lung cancer		[164]
	Skin cancer risk is heightened with atopic dermatitis but decreased with other allergic conditions		[161]
	Atopic dermatitis is protective against different cancers but not protective against cutaneous malignancies		[161, 165]

Several studies report epidemiological links between serum IgE levels and cancer risk. IgE deficiencies have been associated with increased cancer risk, particularly with haematological malignancies, compared with normal IgE serum levels (Figure 1) [24, 25, 37]. Low serum IgE and IgM levels appear to correlate with higher cancer rates, with no associations found for IgA or IgG [37]. Polymorphisms in IL4 and IL4R alleles, both associated with B cell class‐switching to IgE and enhanced serum IgE levels, are linked with gastrointestinal (GI) cancer risk in Caucasians: the IL4 rs2070874 T variant shows positive associations while heterozygous IL4R rs1801275 carriers show negative associations [38]. Other mutations related to IgE metabolism, including DOCK8 deficiency linked to Hyper‐IgE syndrome, appear correlated with increased cancer risk [39], potentially pointing to opportunities to monitor patient groups with mutations known to alter IgE metabolism.

While associations between IgE deficiencies, low serum IgE and IgM levels with an increased risk of the development of some cancers have been reported, there is no evidence of altered levels of serum IgE in cancer. Furthermore, whether cancer antigen‐specific IgE levels are present in healthy populations or patients with cancer is yet to be elucidated.

### 2.1 Glioma: A Paradigm of Epidemiological and Functional Links Between Allergy, IgE and Immune Protection

A history of allergies has been reported to correspond to a 20%–40% lower likelihood of developing glioma [41, 42] the subject of a recent AllergoOncology position paper [32]. Glioma subtypes are defined by molecular features such as isocitrate dehydrogenase (IDH) mutation status, which carries major prognostic implications and the IDH‐wild‐type (IDH‐WT) subtype encompassing glioblastoma (GBM), the most frequent and clinically challenging form [40].

Multiple retrospective and nested case–control studies demonstrate inverse associations of elevated total serum IgE with glioma incidence [43, 44]. Moreover, findings from a smaller GBM cohort linked elevated IgE levels to extended survival of patients diagnosed with this cancer type. These may point to IgE‐driven immune mechanisms contributing not only to tumour prevention but potentially to modulating progression of established disease [45]. In the largest study to date, Guerra et al. [46] examined over 2200 glioma cases using molecularly classified datasets. Higher levels of total and respiratory allergen‐specific IgE were associated with reduced risk of both IDH‐mutant and IDH‐WT gliomas. This protective association was strongest in females with IDH‐WT gliomas. These findings underscore that sex, IgE antigenic specificity and tumour subtype may be linked with allergic immunity and could shape glioma outcomes.

Two studies highlighted the role of polymorphisms in the *EMSY* (*C11orf30*) locus, associated with IgE regulation and atopy, as potential genetic modifiers of glioma risk and prognosis [47, 48]. Notably, higher *EMSY* expression was associated with improved survival in men under 70 years of age with lower‐grade glioma, but this link was not observed in women [48]. In contrast, *EMSY* variants such as rs12278256‐T and rs7130588‐G were linked to increased glioma risk and worse survival in women with IDH‐mutant gliomas but not in men [47]. These may indicate sex‐specific genetic regulation of IgE pathways.

Mechanistically, IgE may influence glioma biology through both peripheral and central nervous system (CNS)–specific pathways. Peripheral allergen exposure can lead to IgE cross‐linking on mast cells (MC) and basophils, releasing neuroactive mediators, including serotonin, histamine and IL‐4, that in turn may modulate brain function via engagement of neural circuits such as the vagus nerve [49]. Concurrently, IgE and related immune cells can cross into the brain parenchyma, where they may activate microglia and potentially enhance immune surveillance [32].

Studies in experimental in vivo models support links between allergic inflammation and glioma progression. Allergic airway inflammation (AAI) was shown to delay glioma progression. In mouse models, asthma‐induced AAI suppressed optic glioma formation via T cell–dependent inhibition of CCL5 secreted by microglial cells [50]. Furthermore, AAI prolonged survival in GBM‐bearing mice by enhancing microglial activation and CD4+ T‐cell infiltration, an effect lost in immunodeficient RAG1‐KO mice [51]. However, the specific contribution of IgE within these contexts remains unclear. Furthermore, as IgE and its FcεR expression and distribution differ significantly between mice and humans, especially with regards to the absence of FcεRI in mouse microglia, monocytes and macrophages, these findings may not fully reflect human IgE immune responses. Despite limitations, collectively, these findings suggest that allergic immune pathways may play more active immunological roles in glioma biology than previously recognised.

Current evidence overall supports further research integrating molecular classification, sex‐specific factors, distinctions between total and allergen‐specific IgE and IgE‐associated genetic variants towards gaining a more nuanced understanding of glioma risk and disease progression. Investigation into allergic markers, including IgE, is warranted, particularly for IDH‐WT gliomas where new stratification and treatment strategies are urgently needed.

## 3 Immune Cells and Their Mediators Known to Participate in Allergic Responses Hold Clinical Significance in Cancer

**FIGURE 2:**
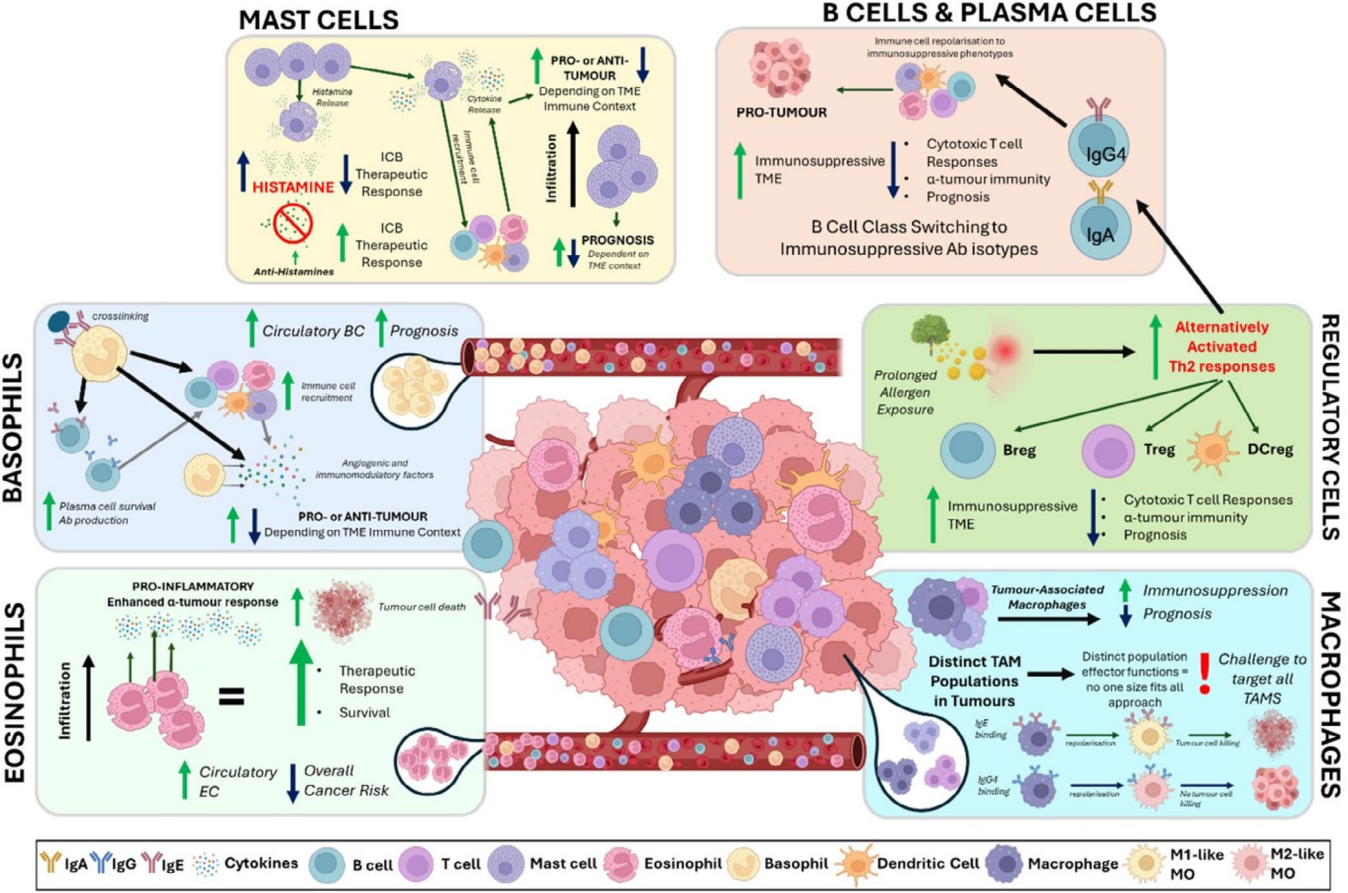
Roles of major allergy‐associated immune cells in tumour biology [21, 36, 74, 75, 101, 102, 103, 105, 159]. A complex interplay of interactions between allergy‐associated immune cells and the tumour microenvironment dictates the tumour response to the presence or absence of these cell types. The release of granules by allergy‐associated cells can have a highly contextual impact on the TME, depending on the inflammatory state of the tumour. Classical Th2 immune responses associated with allergic response, such as IgE production, granulocyte and dendritic cell activation also promote anti‐tumour immune responses whilst alternatively activated Th2 responses can lead to the activation of regulatory immune cells including Tregs and Bregs, potentiating a tumour‐permissive immunosuppressive environment. Immune cell infiltration may impact outcomes through the release of cytokines and recruitment of additional immune cell populations. Some tumour‐associated cell populations, such as tumour‐associated macrophages, are consistently associated with poor prognosis and are challenging to target. Consideration of the immune context of the tumour, including immune cell infiltrates, may allow for patient stratification and development of novel therapies. Created with BioRender.com. Ab, antibody; BC, basophil count; Breg, regulatory B cell; DCreg, regulatory dendritic cell; EC, eosinophil count; ICB, immune checkpoint blockade; TAM, tumour‐associated macrophage; TME, tumour microenvironment; Treg, regulatory T cell.

Immune cells known for multifaceted roles in allergy may contribute to pro‐ or anti‐cancer immunity. The complexity and heterogeneity of contributions to allergic mechanisms of cells associated with allergy, such as granulocytes (eosinophils, basophils, neutrophils), MC and monocytes/macrophages, are also reflected in their roles within cancer [52] (broadly summarised in Tables 3 and 4). Evidence indicates both prognostic significance of the presence or absence of these cells, their subsets and the inflammatory mediators they may secrete within the tumour microenvironment (TME) and across the body, as well as putative anti‐tumour functions that may be harnessed for therapy (Figure 2).

**TABLE 3:** The main characteristics and functions of FcεR‐expressing immune cells in allergy and cancer.

Cell type	Role in allergy	Role in cancer	Prognosis	Current/future work	References
Mast cells	Express tetrameric αβγ2 FcεRI IgE‐sensitised Release histamine, cytokines (IL‐4, IL‐13) Promote inflammation, itch, bronchoconstriction	Secrete mediators including TNF‐α, VEGF, PDGF‐β, IL‐6, IL‐1, TGF‐β, IL‐10 Contribute to tumour growth, angiogenesis, and immune modulation Regulate the TME by recruiting or influencing T cells, NK cells, macrophages, neutrophils, MDSCs, and dendritic cells Exert pro‐ or anti‐tumour effects depending on receptor interactions (e.g., histamine and TNF‐α) Modulate immune responses by suppressing Tregs, inhibiting T cell activation via PD‐L1, or promoting tolerance	Favourable in oesophageal adenocarcinoma, ovarian cancer, and diffuse large B‐cell lymphoma Poor/Mixed in gastric, lung, melanoma, and breast cancers Prognostic significance depends on localization	Ongoing studies explore repurposing allergy treatments Promising results in combinations with immune checkpoint blockade Future work will focus on patient tissue‐based translational studies and rational clinical trial design to optimise combination strategies and monitor therapeutic efficacy and safety	[58, 59, 60]
Eosinophils	Expression of trimeric αγ2 FcεRI remains controversial Recruited by IL‐5, IL‐4 Release cytotoxic granules Key in airway hyperactivity and tissue remodelling	Infiltrate various tumour types (e.g., melanoma, colorectal, breast and bladder cancers) Exert antitumour properties through direct cytotoxicity, ADCC, and production of proinflammatory cytokines Enhance recruitment and activation of CD8^+^ T cells and NK cells by secreting chemokines such as CCL5 and CXCL9/10 Contribute to vascular normalisation and facilitate T cell infiltration in tumours May shift towards tumour‐promoting functions in certain settings (e.g., in IL‐13–rich environments)	Favourable prognosis; in melanoma, colorectal cancer, bladder cancer, and some lung cancers Eosinophil infiltration often correlates with improved response to immunotherapy (e.g., anti‐PD‐1) and better overall survival Associated with tumour invasion and worse overall survival in some cases	Under investigation as potential enhancers of cancer immunotherapy Eosinophil‐boosting strategies are being tested in preclinical models to improve CD8^+^ T cell infiltration and therapy response Further work is needed to define their role in different TMEs and to harness their cytotoxic and immunomodulatory capabilities in therapeutic contexts	[62, 76, 77]
Basophils	Express tetrameric αβγ2 FcεRI Release IL‐4, IL‐13, LTC4 Contribute to Th2 skewing and pruritus	Recruit and activate other immune cells such as T cells and B cells Produce angiogenic factors (VEGF) and immunomodulatory molecules that can either promote or inhibit tumour progression depending on context Activation of basophils may enhance type 2 immune responses and M2 macrophage polarisation Sustain plasma cell survival and antibody production, contributing to anti‐tumour immunity in some solid tumours	Favourable prognostic markers in melanoma, ovarian cancer, colorectal cancer, NSCLC (particularly with immune checkpoint inhibitors), and glioblastoma Unfavourable or associated with poor outcomes in myeloid malignancies (PV, CML, MDS), prostate, bladder, and gastric cancers	Therapeutic targeting of basophil‐mediated pathways (e.g., histamine receptors, cytokines) is underexplored but may complement checkpoint blockade or antibody therapies Understanding basophil plasticity and recruitment mechanisms remains a priority to exploit their anti‐ or pro‐tumour potential effectively	[61, 62]
Dendritic cells	Express trimeric αγ2 FcεRI Present allergens to naive T cells Promote Th2 differentiation and IgE responses Promote Tregs generation	Cross‐presentation of tumour‐associated antigens Promote CD8+ T cell immunity Support Th1 polarisation; produces cytokines (IL‐12, type I IFNs) to enhance anti‐cancer activity of T cells Produce CXCL9, CXCL10 chemokines to recruit T cells Express co‐stimulatory molecules to enhance T cell priming and survival	High cDC1 density correlates with better prognosis in breast, ovarian, prostate cancers, head and neck squamous cell carcinoma and melanoma Improves response to anti‐PD1 Unfavourable prognosis in some cases, including melanoma and oral squamous cell carcinoma	Therapeutic vaccination with cDC1 Boost cDC1 recruitment and survival (e.g., via FLT3L) Combine DC vaccines with checkpoint inhibitors Develop strategies to reverse DC tolerance Enhance DC recruitment and function	[166]
Macrophages	Express trimeric αγ2 FcεRI and FcεRII following stimulation with IL‐4 M2 phenotype induced by IL‐4/IL‐13 Promote chronic inflammation and tissue remodelling Recruit type 2 immune cells	Secrete IL‐6, IL‐1β, TNFα to promote tumour‐promoting inflammation Induce EMT and ECM remodelling to aid tumour metastasis Secrete VEGF, MMPs, PDGF to promote vessel growth and permeability Release IL‐10, TGFβ and expressed PD‐L1 to suppress immune activity	High CD163+ macrophage abundance correlates with poor prognosis in various solid tumour, including lung, colorectal, gastric, liver, and breast cancer	Targeting TAMs by depletion or inhibiting recruitment Repolarization to M1 phenotype to macrophage‐mediated phagocytosis Development of macrophage‐targeting drugs including small molecules, antibodies, nanoparticles, CAR‐macrophages	[68, 69]
IgE^+^ B cells	Produce allergen‐specific IgE; central to sensitization and allergic effector responses	Natural immune surveillance Activate unique effector cells induces pro‐inflammatory macrophages IgE mediates ADCC/ADCP via FcεRI and CD23 Re‐educate TAMs Promote DC cross‐presentation	High IgE levels correlated with reduced risk of glioma, chronic lymphocytic leukaemia, and multiple myeloma	MOv18 IgE Phase 1 trial completed MOv18 IgE Phase 1b trial ongoing Pre‐clinical evaluation of efficacy and safety for other IgE‐based therapies	[167, 168]

Abbreviations: ADCC, antibody‐dependent cell‐mediated cytotoxicity; ADCP, antibody‐dependent cell‐mediated phagocytosis; CML, chronic myelogenous leukaemia; DC, dendritic cells; MDS, myelodysplastic syndrome; MMP, matrix metalloproteinase; NSCLC, non–small‐cell lung cancer; PV, polycythaemia vera; TAM, tumour‐associated macrophage; Th1, T‐helper 1; VEGF, vascular endothelial growth factor.

**TABLE 4:** The main traits and functions of non‐FcεR expressing immune cells in allergy and cancer.

Cell type	Role in allergy	Role in cancer	Prognosis	Current/future work	References
CD4+ helper type 2 (Th2) cells	Produce type 2 cytokines such as IL‐4, IL‐5, IL‐13 Drive IgE class switching Promote allergic inflammation	Protective in some tumours via cytokine production Chronic inflammation in atopy may promote tumorigenesis Polymorphisms in IL‐4 gene linked to variable cancer risk May drive tumour elimination in some models Infiltration linked to immune escape and cancer proliferation in others	Good prognosis linked to GATA‐3 and c‐Maf expression in breast cancer, Hodgkin lymphoma Poor prognosis in gastric, pancreatic, neuroblastoma, lung adenocarcinoma, adrenocortical, kidney cancers No clear prognostic value in glioblastoma, NSCLC, melanoma, breast, colorectal cancers	Combined T cell and innate immune cell therapies Eosinophil and Th2 adoptive cell transfer Targeting IL‐4 receptor and TGF‐β receptor to block detrimental Th2 effects Reactivation of tumour‐infiltrated Th2 cells Nutrient replenishment to restore Th2 function IL‐4 receptor inhibition combined with radio/chemotherapy	[62, 169]
ILC2s	Innate source of IL‐5, IL‐13 Respond to IL‐33 Amplify type 2 responses	Secret IL‐33 that activates stromal and immune cells creating pro‐tumour microenvironment PD‐1+ ILC2s promote tumour growth via immune checkpoint pathways Recruit immunosuppressive neutrophils, MDSCs and Tregs Lung ILC2s linked with metastasis via eosinophil‐mediated NK cell inhibition IL‐33‐activated ILC2s recruit DC and enhance CD8+ T cell anti‐tumour response in PDAC	High ILC2 levels often correlate with worse outcomes (HCC, breast, lung), but sometimes better survival (CRC) ILC2:ILC1 ratio and PD‐1+ ILC2 subsets affect prognosis	Ongoing studies aim to elucidate mechanisms behind ILC2‐mediated tumour promotion and suppression Investigate targeting IL‐33/ILC2 signalling and PD‐1+ ILC2s Explore therapeutic strategies harnessing or inhibiting ILC2 functions to improve cancer treatment	[170, 171]
CD8^+^ T cells (Tc1)	Limited role Cytotoxicity downregulated in allergic inflammation	Produce perforin, granzyme B, IFN‐γ, and TNF‐α to eliminate tumour Highly infiltrate in immune‐active tumours High level of immune checkpoint molecules in TME lead ot Tc1 exhaustion	High abundancy of Tc1 cells in cancer patients is associated with improved survival and better ICI responses in many cancers, including colorectal, renal, breast, ovarian, pancreatic and gastric cancers However, poor prognosis was observed in renal cell cancer	More than approved 7 ICI therapies and 7 CAR‐T cell therapies Combinations of ICI with other immunotherapies could improve the therapeutic outcome	[172, 173]
Type 2 CD8^+^ cells (Tc2)	Produce IL‐4, IL‐5, IL‐13; enhance humoral immunity Recruiting eosinophils Amplify allergic inflammation Involved in steroid‐resistant allergy and chronic inflammation	Reduced cytotoxicity Promote Th2 inflammation and tumour immune evasion	High abundancy of Tc2 cells in cervical cancer and urothelial bladder cancer correlated with immune escape	Redirect allergy therapies which target Tc2 populations against tumours	[174, 175]
Regulatory T cells (Tregs)	Suppress Th2, Th1, and Th17 responses via IL‐10, TGF‐β, IL‐35 Maintain immune tolerance Reduced or dysfunctional in allergy	Release granzyme B and perforin to induce T effector cells apoptosis and cytolysis Release TGF‐β, IL‐10 and IL‐35, which suppress T effector cells function and mediate T cell exhaustion Increased expression of immune checkpoint molecules Express ectoenzymes to induce adenosine‐mediated immunosuppression	Poor prognosis in colorectal, head and neck, lung, renal, gastric, breast, ovarian, pancreatic, and prostate cancer However, improved prognosis in immune‐active tumour by suppressing pro‐tumour inflammation	Further studies in markers for Tregs subgroups in TME Investigation of Treg differentiation Development of immune checkpoint blocking strategies for Tregs in combination with other anti‐cancer therapies	[176, 177]
Neutrophils	Release mediators like myeloperoxidase (MPO) and platelet‐activating factor (PAF), which intensify inflammation and further activate mast cells Can be directly stimulated by allergen‐IgG complexes via Fcγ receptors, independent of mast cells	Immune suppressive (MDSCs) promote tumour cell dissemination Pro‐inflammatory signals via NETosis Promote angiogenesis Induce EMT for tumour cell motility Trigger dormant metastatic cells via NETs Kill opsonized tumour cells via trogocytosis Present tumour antigens to T cells Neutrophil‐derived TRAIL kills tumour cells Increased neutrophil counts predict immunotherapy response	Poor prognosis in mesothelioma, pancreatic cancer, renal cell carcinoma, colorectal carcinoma, gastroesophageal cancer, non–small cell lung cancer, cholangiocarcinoma, and hepatocellular carcinoma Ly6Ehi neutrophils predict anti‐PD1 response and sensitise tumours to anti‐PD1 in melanoma and lung cancer HLA‐DR+ antigen‐presenting neutrophils correlate with survival upon immune therapy	New anti‐neutrophil therapy needed for tumour environment Identify novel neutrophil subtypes using scRNA‐seq; scRNA‐seq and pseudo‐time analysis for predicting outcomes and targeting differentiation Consensus on N‐MDSC types and markers needed No direct neutrophil‐specific inhibitors available	[178, 179, 180]

Abbreviations: CAR‐T, chimeric antigen receptor T cells; EMT, epithelial‐mesenchymal transition; MDSC, myeloid‐derived suppressor cells; NET, neutrophil extracellular trap; Th1, T helper 1.

Histamine, the most widely described allergic mediator primarily secreted by mast cells and basophils in allergic reactions and inflammation, has been reported to participate in cancer inflammation, including through interactions with its receptors (e.g., H1 (HRH1)). A study reported that histamine can exert direct antitumor effects in a mouse model of triple‐negative breast cancer by reducing tumour cell proliferation and enhancing apoptosis, while also modulating the immune response by increasing cytotoxic T cell infiltration and decreasing immunosuppressive cell populations [53]. These findings suggest that histamine, through its immunomodulatory and cytotoxic actions, may serve as a potential therapeutic agent or adjuvant in breast cancer treatment strategies. In contrast, a clinical study found higher serum histamine levels in non–small‐cell lung cancer patients compared with a cohort diagnosed with benign pulmonary nodules and in the patient group elevated histamine levels were associated with tumour metastasis [54]. Activation of HRH1 has also been shown to promote hepatocellular carcinoma cell growth [55]. In an orthotopic mouse model of pancreatic ductal adenocarcinoma expression of HRH1 and signalling within tumour cells could suppress MHC‐I expression, impairing antigen presentation to cytotoxic T cells [56]. Inhibition of HRH1 restored MHC‐I levels, improved T cell recognition and significantly boosted the antitumor effects of ICI. Histamine thus appears to exert a complex and context‐dependent role in cancer, exhibiting both antitumour and tumour‐promoting effects through its receptor‐mediated actions.

Beyond histamine, allergy effector cells release a wide array of mediators that may influence tumour development and progression through multiple mechanisms [14, 52, 57, 58, 59, 60, 61, 62]. MCs secrete proteases tryptase and chymase, proteoglycans (e.g., heparin) and a wide range of cytokines and chemokines, growth factors and angiogenic mediators. These have the capacity to remodel the extracellular matrix, increase vascular permeability, stimulate neo vasculature formation and recruit or polarise myeloid populations. These can shape the TME to either support tumour growth and invasion or enhance anti‐tumour immunity [58, 59, 60].

Lipid mediators released by activated MCs and basophils, notably prostaglandins and leukotrienes, are known to regulate inflammation, vascular tone and immune cell trafficking and can promote tumour cell proliferation, survival and metastatic behaviour in certain settings [58, 59, 60, 61, 62, 63]. Proteases activate matrix metalloproteinases and cleave extracellular matrix components, facilitating invasion and dissemination while also generating peptide fragments that may trigger immune recognition [64]. Charged granule components such as heparin influence coagulation and tumour cell–platelet interactions relevant to metastatic seeding [65]. Cytokines (e.g., IL‐5, IL‐4, IL‐13), chemokines (e.g., eotaxin‐1 [CCL11], eotaxin‐2 [CCL24] and eotaxin‐3 [CCL26]) and monocyte chemotactic proteins (MCPs), secreted by allergy effector cells, recruit eosinophils, macrophages and monocytes and shape macrophage polarisation [14, 66, 67]. Type 2 cytokine–driven M2‐like macrophage phenotypes promote immunosuppression and tissue remodelling, whereas proinflammatory mediators can stimulate antigen presentation and immune activation [14, 62, 68, 69].

Granulocytes and MC commonly associated with the allergic/atopic response are also known to participate in cancer inflammation [52, 57]. Eosinophils hold prognostic value across a wide range of cancers, including colorectal (CRC) [70], ovarian [71], prostate [70], hepatocellular carcinoma [70], advanced upper GI cancers [72] and GBM [70, 73]. Higher absolute eosinophil count (EC) in blood was associated with both lower overall cancer risk and lower risk of specific cancer types, including melanoma, breast and chronic lymphocytic leukaemia [74]. This includes GBM, where increased preoperative circulatory eosinophil levels appear correlated with improved long‐term survival [75]. These suggest protective roles in anti‐tumour surveillance [74]. Eosinophils release major basic protein, eosinophil cationic protein and eosinophil peroxidase and form extracellular traps; these can exert direct cytotoxicity against tumour cells, modulate local inflammation and influence fibroblast and vascular responses, with tissue eosinophilia showing variable associations with clinical outcome across tumour types [62, 76, 77]. Eosinophil‐based indices in patient blood and tumours may hold prognostic value not just for cancer outcomes but for therapeutic response to ICI, including in the neoadjuvant setting [70, 75, 78, 79]. Monitoring of eosinophil indices may therefore provide insight into progression, prognosis and patient stratification for therapeutic response.

Basophils have long‐standing associations with mediating powerful allergic effector functions [70]. Whilst predominantly circulatory, increasing evidence indicates their presence in both normal tissues [80, 81] and tumour lesions [70, 73]. The roles of basophils in the TME appear highly contextual and may depend on local inflammatory milieu [70]. Basophils produce histamine, cysteinyl leukotrienes and type 2 cytokines that may alter dendritic cell function and T cell priming, potentially skewing adaptive responses towards Th2 phenotypes that may suppress cytotoxic T cell activity or alternatively enhance antibody‐dependent mechanisms [61, 62]. Basophil indices, including circulatory basophil count (BC), hold prognostic value across a wide range of cancers [70, 73, 82, 83, 84]. Alongside their use in assays such as the Basophil Activation Test (BAT) for identification and monitoring of hypersensitivity reactions (see below), monitoring of basophils may represent a valuable tool for predicting clinical outcomes.

FcεRI‐expressing MC, which can be stimulated by IgE‐allergen immune complexes, can exert both pro‐ or anti‐tumorigenic activities. These functions depend on tumour type, tumour anatomical location and immunological context of the TME [52]. Correspondingly, levels of MC tumour infiltration may have either positive [85, 86, 87, 88] or negative [82, 89, 90, 91, 92] prognostic significance. MC infiltration may also be prognostic of decreased response to ICI across various cancers [82, 89, 93], potentially linked to roles for histamine in modulating ICI [36]. MC infiltration may represent an important index for therapeutic stratification, particularly in non‐responders to ICI who may benefit from therapies targeting histamine or MC. Furthermore, it may be possible in a therapeutic setting to polarise MC towards activatory states that allow them to mediate anti‐tumour functions in the TME [94].

Neutrophils are highly abundant in circulation but can be recruited to tumour lesions, where their maturation, activation and survival are highly influenced by the TME. Tumour‐infiltrating neutrophils may promote cancer cell growth, metastasis, angiogenesis, tissue necrosis through vascular occlusion, alongside supporting immunosuppression, including through neutrophil extracellular trap (NET) formation, which is normally formed as part of pathogen immune responses, to instead support tumour progression [95, 96, 97, 98]. The formation of NETs in the TME is reported to predict disease progression across different cancers [96, 97]. In the circulation, neutrophil‐to‐lymphocyte ratios have also been reported to associate with less favourable clinical outcomes [96, 97].

Macrophages are less well described in allergy, but express FcεRs and are well described to infiltrate tumour lesions at high densities. The recruitment, activation and phenotypes of these cells are influenced by a variety of immunosuppressive signals in the TME [14]. Tumour‐associated macrophages (TAMs) are largely polarised towards immunosuppressive (M2‐like) phenotypes, influenced by hypoxic conditions and alternative Th2‐skewed inflammatory mediators such as IL‐4, TGF‐β and IL‐10 [14]. Therapeutic approaches to either deplete, reduce or reprogramme these cells towards pro‐inflammatory states have largely failed as a therapeutic avenue and the presence of TAMs is generally associated with poorer prognosis [99, 100]. Future insights into the presence and localisation of TAM populations with unique functions and gene signatures within the TME may offer new therapeutic avenues for targeting or harnessing TAMs [99, 100]. Therapeutic approaches may entail employing tumour antigen‐specific IgE antibodies to cross‐link FcεRs expressed on macrophages in the presence of multivalent antigens expressed by cancer cells in the TME, potentiating macrophage stimulation, repolarisation and effector functions, described further below [14].

## 4 Insights From Allergic Inflammation to Immune Tolerance in Cancer

In allergy, the immune system responds to largely innocuous substances, allergens, through the induction of classical Th2 pathways that drive B cells to class‐switch to IgE. While induction of allergic mechanisms may exert protective anti‐tumour effects, prolonged antigen exposure can result in alternatively activated Th2 responses which can promote cancer progression. This Th2 inflammatory response results in the induction of regulatory subsets of dendritic cells (DCregs) [101], T cells (Tregs) and B cells (Bregs) (Figure 2) [102, 103]. These, in turn, help to promote an immunosuppressive TME, resulting in impaired anti‐cancer cytotoxic T cell responses and poor clinical prognosis. ICIs targeting PD‐1 and CTLA‐4, negative regulators of T cell activation, promote cytotoxic T cell responses to break immune tolerance via blockade of these pathways [34, 104, 105]. The mechanisms by which tumours bypass classical IgE‐driven response to induce alternatively activated Th2 immunity in cancer leading to immune tolerance remain to be fully elucidated. These insights may reveal avenues to moderate allergic inflammation as well as mechanisms to stimulate anti‐cancer cascades in AllergoOncology.

Immune tolerance is also influenced by the composition of the gut microbiome, which has been linked to cancer patient outcomes and ICI response [106, 107]. A favourable gut microbiome has been associated with better responses to anti‐PD‐1 treatment in melanoma patients [106]. Microbial communities in the gut and the TME shape local and systemic anti‐tumour immunity by influencing antigen presentation, dendritic cell and T cell priming and myeloid cell phenotypes [109, 110, 111, 112]. Limited exposure to potential allergenic agents early in life, limited heterogeneity of the gut microbiome and lower abundance of gut microbes are linked to increased risk of atopic disease [113, 114], and deficiency in regulatory immune cell populations [114].

Dysbiosis of the gut microbiome can drive chronic inflammation and impair mucosal barrier integrity, altering drug delivery and immune cell infiltration and creating a pro‐tumour environment [108]. Antibiotic usage has been shown to inhibit response to anti‐PD‐1 therapy in both mouse models and cancer patients [107], with the resulting dysbiosis correlated with allergy [115]; however, in other cancers such as pancreatic ductal adenocarcinoma, antibiotic usage has been shown to improve outcomes [116]. The impact of antibiotic usage may be determined by the composition of tumour‐resident bacteria, some of which may be able to metabolise and inactivate chemotherapeutic agents such as gemcitabine and thus promote chemoresistance. Depletion of these microbes can restore or enhance drug efficacy and has been associated with improved survival in some cohort studies [116, 117].

## 5 Harnessing IgE Class Antibodies for Cancer Therapy

All currently available therapeutic monoclonal antibodies belong to the IgG class, of which IgG1 is the predominant isotype [118]. However, there is growing interest in the development of antibodies engineered using alternate antibody scaffolds including different isotypes such as IgE and IgA, which possess effector mechanisms and functions distinct to IgG. IgE‐based antibodies for cancer therapy are part of this growing area of research and development [12, 20, 21, 119]. IgE is the predominant antibody class known for its roles in the allergic response and for immune‐stimulating functions against tissue‐resident parasites [118]. Several potent immune‐stimulating properties are attributed to IgE, which may be applied for cancer therapy. Through high affinity FcεRI:IgE interactions, surpassing those of IgG:Fcγ receptor affinities by 100–10,000 fold [118], IgE can activate a wide range of immune effector cells expressing cognate FcεRs (FcεRI and FcεRII/CD23), including macrophages, MC, eosinophils, basophils and dendritic cells [19, 73, 119, 120]. The high affinity of IgE for and slow dissociation from FcεRs on these cells can translate to prolonged tissue retention including in tumours, many of which feature FcεR‐expressing immune cell population infiltrates (e.g., macrophages, MC) [121, 122]. Furthermore, IgE has no known inhibitory Fc receptors [118]. IgEs directed to tumour‐associated antigens therefore may exert anti‐tumour mechanisms different to those of existing IgG‐based therapeutics through engagement of distinct effector cell populations, robust FcεR:IgE interactions and heightened or prolonged tissue immune surveillance [21]. These attributes and well‐described potency of IgE antibodies in allergic diseases have sparked interest in the generation and application of anti‐tumour IgEs as a novel anti‐cancer therapeutic modality [18, 21, 119, 123, 124].

### 5.1 Evidence for Anti‐Tumour Mechanisms of Recombinant IgE Antibodies Directed Against Cancer Antigens

Alongside epidemiological evidence linking IgE to decreased cancer risk for some malignant indications [13, 15, 24, 37], studies highlight anti‐tumour efficacy of tumour antigen‐specific IgEs both in vitro and in vivo [18, 21, 119, 123, 124]. Several recombinant IgE antibodies have been developed, specific for a range of tumour‐associated antigens including the epidermal growth factor receptor 1 (EGFR) [125], prostate‐specific antigen (PSA) [126], mucin 1 (MUC1) [124], CD20 [124] and CD38 [18]. Across different malignant indications, these agents have demonstrated Fc‐mediated anti‐tumour functions including recruitment and stimulation of immune cells to mediate cancer cell killing, restriction of tumour growth and TME reprogramming towards a pro‐inflammatory, anti‐tumour state [19, 21, 119, 121]. Preclinical studies comparing the anti‐tumour activities of tumour‐directed IgE antibodies against their corresponding tumour antigen‐specific IgG isotypes showed evidence of superior efficacy across different human tumour xenograft models in mice engrafted with human immune cells, as well as in immunocompetent syngeneic rat tumour models [127, 128, 129, 130].

Two emerging IgE antibodies, HER2‐IgE and CSPG4‐IgE, are directed against the human epidermal growth factor receptor 2 (HER2) expressed in subsets of breast cancer and ovarian carcinomas and chondroitin sulfate proteoglycan 4 (CSPG4), overexpressed in melanoma, basal breast cancers and other malignancies, respectively. These antibodies have demonstrated Fc‐mediated functions against antigen‐expressing cancer cells, including ADCC/ADCP activity and MC degranulation [119, 121, 123, 131, 132, 133]. IgE formats engineered with the variable regions of the clinically approved anti‐HER2 antibody trastuzumab can exert Fab‐mediated direct effects suppressing cancer cell proliferation and disrupting HER2 signalling pathways, indicating that IgE retains Fab‐mediated mechanisms of the corresponding IgG1 therapeutic agent [121, 123, 131, 132, 133]. In vivo studies of HER2‐IgE demonstrated prolonged survival of tumour challenged mice, tumour growth inhibition and significant changes to the TME promoting a pro‐inflammatory anti‐tumour environment [131, 133]. Similarly, anti‐tumour efficacy was demonstrated by CSPG4‐IgE: in vivo studies showed significant tumour growth restriction and extended survival in autologous patient‐derived immune cell‐and tumour cell‐engrafted xenograft models of melanoma, along with increased infiltration of monocytes and macrophages and activation of pro‐inflammatory pathways [119, 120].

The most extensively studied recombinant IgE, MOv18 IgE recognises the tumour‐associated antigen folate receptor alpha (FRα), expressed in several solid tumours including ovarian and triple‐negative breast cancers. MOv18 IgE stimulates ADCC/ADCP by human immune cells, predominantly monocytes and macrophages and triggers MC degranulation and killing of FRα + cancer cells [128, 129, 130]. MOv18 IgE can stimulate and recruit monocytes/macrophages against cancer cells via activation of a TNF/MCP‐1 signalling axis, triggered by crosslinking of FcεRI‐MOv18 IgE complexes by multiple copies of FRα expressed on the surface of tumour cells [127]. These interactions initiated by IgE trigger significant pro‐inflammatory stimulation signals in vitro and in animal models of cancer [127] and likely contribute to the superior anti‐tumour efficacy of MOv18 IgE in comparison with a corresponding anti‐FRα IgG in preclinical studies.

MOv18 IgE is shown to engage immunosuppressive ovarian cancer patient–derived macrophages, repolarising them to hyperinflammatory, T cell‐stimulatory phenotypes, able to limit Treg induction and promote CD8^+^ T cells. This IgE‐driven pro‐inflammatory signature is linked to more favourable prognosis in ovarian cancer [14]. High‐dimensional flow cytometry and RNA‐seq studies identified increased infiltration of CD68^+^ macrophages and CD3^+^ T lymphocytes in tumours from animals which received MOv18 IgE. These findings underscore a significant attribute for IgE in potentiating a global immunological shift towards enhanced anti‐tumour immunity.

MOv18 IgE is the first IgE‐based therapeutic to progress to a first‐in‐human Phase 1 clinical trial (NCT02546921) in patients with FRα + gynaecological cancers [21]. This trial was successfully completed, demonstrating a favourable safety profile for IgE and early evidence of efficacy [21]. In concordance with findings in pre‐clinical studies, treatment of patients with MOv18 IgE resulted in enhanced macrophage and T cell recruitment into tumours, further supporting the notion that IgE immunotherapy has the potential to harness the patient's immune cells. MOv18 IgE has progressed to a Phase 1b study (NCT06547840) in platinum‐resistant ovarian cancer patients, with recruitment currently open.

Collectively, these highlight the potential of IgE‐based therapeutics to directly target immune effector cells against cancer, but also to modulate the TME towards immunoactive states. Inspired by IgE‐driven allergic responses, IgE class antibodies may thus offer a promising avenue for cancer treatment.

### 5.2 Development of Engineering Platforms for Generating Therapeutic IgE Antibody Candidates for Cancer Therapy

Exploration of therapeutic IgEs has mandated the efficient cloning, production and purification of IgE class antibodies at sufficient yields and purity for preclinical efficacy and mechanism of action studies. Research‐grade recombinant IgE (rIgE) has been successfully generated in different expression systems including plants [132], human (Expi293F) [120, 123, 134] and rodent (SP2/0, CHO) cells [135]. MOv18 IgE is the first rIgE to be generated at a clinical grade suitable for human administration. The inaugural process development and Good Manufacturing Practice (GMP) production of this antibody has overcome previously perceived notions of limitations in manufacturing recombinant IgE for clinical use. Recent work demonstrated the successful and scalable production of MOv18 IgE at a GMP standard suitable for administration in humans, including high purity, good stability, as well as retention of biophysical characteristics, glycosylation and functional profiles comparable to research grade antibody [135, 136]. This yielded the first IgE Investigational Medicinal Product (IMP) and laid the foundation for the development of IgE as a new class of anti‐cancer antibodies.

Generation of new antibodies engineered with IgE Fc domains can take advantage of classical pipelines typically employed for engineering conventional IgG antibodies, such as phage display technology. A platform for the selection of lead candidates recognising HER2 enabled the selection and development of HER2‐IgEs engineered with human and rat IgE Fc domains that exhibited potent immune‐stimulating and tumour‐restricting properties in vitro and in vivo [133]. Alongside, a new IgE class‐specific affinity matrix was tested for the rapid and efficient production of high‐purity IgE antibodies with human Fc domains [137]. These support the wide applicability of universal pipelines in the generation of high‐quality and functional IgG towards production of recombinant IgE.

Other formats such as bispecific antibodies may offer superior tumour‐targeting specificity, minimising the likelihood of off‐tumour toxic effects [138]. IgE bispecific antibodies have demonstrated favourable functional profiles and enhanced anti‐cancer cytotoxicity compared to IgG counterparts in preclinical models [138]. A new hybrid IgE‐IgG1 (IgEG) construct was reported as a novel anti‐HER2 therapeutic [139]. IgEGs demonstrated dual functionality as IgG and IgE isotypes in vitro alongside unique pharmacokinetics and anti‐tumour efficiency (comparable to IgE) in vivo, underscoring a promising and innovative therapeutic anti‐cancer modality [139].

Platform technologies and innovative IgE‐based antibody formats therefore pave the way for expanded applications of IgE in cancer therapy, and across different malignant indications. These advances may aid the development of novel and highly efficacious IgEs for cancer therapy to the success of IgG‐based biologics.

## 6 The BAT From the Allergy Clinic to Oncology to Evaluate Hypersensitivity Risk to IgE Immunotherapy and Biological Drugs

The fields of allergy and cancer converge in the management of drug hypersensitivity reactions (DHR) to cancer therapies, including chemotherapeutic agents, antibodies and small‐molecule inhibitor drugs. Reactions range from mild cutaneous toxicities to life‐threatening systemic anaphylaxis in some patients. DHR can result in treatment discontinuation or withdrawal, depriving patients of potentially life‐saving therapies [140, 141, 142]. Monitoring and risk evaluation are therefore essential to guide clinical decision‐making and optimise therapeutic access for as many patients as possible.

**FIGURE 3:**
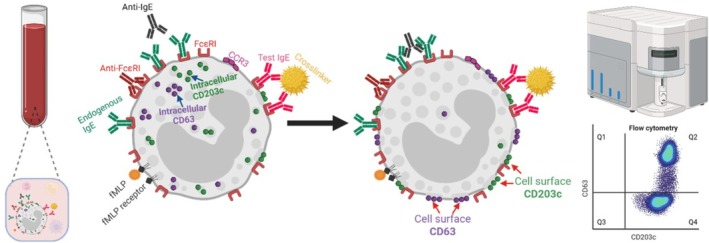
Principles of the basophil activation test (BAT) [70, 160]. Resting basophils contain intracellular vesicles which include CD63 and CD203c. In the BAT, basophils can be activated by IgE‐mediated stimuli such as polyclonal anti‐IgE antibodies cross‐linking cell surface–bound IgE antibodies, or directly via anti‐FcɛRI. Basophil activation can also be stimulated by non‐IgE stimuli such as complement anaphylatoxins or fMLP, a synthetic analogue of a bacterial peptide. Basophil activation results in sequestering of CD63 and CD203c to the cell surface which is measured in the BAT by flow cytometric analysis. Created with BioRender.com.

The BAT is an ex vivo assay applied in the diagnosis of allergies, to confirm type I hypersensitivity. Conducted in whole unfractionated blood, hypersensitivity is measured by the upregulation of activation markers (e.g., CD63, CD203c) on the surface of peripheral blood basophils following stimulation with a suspected allergen or drug [70]. The principle of the BAT is that following ex vivo allergen stimulation, allergen‐specific IgE or any other components on the basophils or in human blood will trigger basophil activation and degranulation, detected by upregulation of activation markers on the basophil surface (Figure 3). The BAT therefore allows detection of suspected type I hypersensitivity against particular agents in as little as a few minutes. Since it is performed in blood samples ex vivo, potential for further sensitisation or systemic reactions is avoided and patient risk minimised, rendering the BAT highly advantageous in high‐risk individuals.

These principles are applied for the use of BAT in patients with DHR against cancer therapeutics. In cancer patients with confirmed DHR, the BAT can then support monitoring of desensitisation protocols designed to induce tolerance to the drug, thus allowing treatment continuation [142, 143, 144]. The BAT may represent a key tool for identifying and monitoring hypersensitivity reactions to IgE immmunotherapy [140, 144, 145], for which we developed a modified BAT protocol to validate patient basophil reactivity against IgE‐mediated (e.g., anti‐FcɛRI, anti‐IgE) and non‐IgE (e.g., fMLP) stimuli [73, 146] and assess basophil activation following ex vivo stimulation with recombinant anti‐tumour IgE using CD63 expression upregulation on the basophil surface as a readout of hypersensitivity propensity to the anti‐tumour IgE. Notably, the BAT conducted before treatment predicted the one patient who experienced a type I hypersensitivity reaction to MOv18 IgE during the first‐in‐human clinical trial [21].

Derived from acquired knowledge and clinical experience in studying allergies, the BAT is translated to oncology and offers a practical, minimally invasive tool that supports personalised care through hypersensitivity risk stratification and monitoring of patients with cancer. This provides an example of the interphase between allergy and oncology to help improve drug safety, preserve or restore patient access to treatment, and thus advance precision medicine in the field of AllergoOncology.

## 7 Linking Allergy and Cancer in AllergoOncology to Explore Therapeutic Avenues and Opportunities

Biological and translational insights derived from AllergoOncology can help shape novel approaches for cancer treatment and inform the management of immune‐related adverse events (irAE), particularly allergic/anaphylactic reactions. Toxicity can greatly restrict the clinical application of cancer immunotherapies and may preclude their continuation [7, 147]. Rapid drug desensitisation has been used to address DHR against chemo‐ and immunotherapies [148, 149]. Additionally, antihistamines, decongestants and corticosteroids are prescribed to manage hypersensitivity symptoms.

Histamine is reported to contribute to both an immunosuppressive TME and to anti‐tumour mechanisms. Consistent with some of these findings, antihistamine administration was shown to enhance therapeutic responses to ICI and significantly improve survival [35, 36, 52]. Similarly, the anti‐IgE therapeutic antibody omalizumab, used in the treatment of allergic and atopic diseases, has been used in the management of various irAE associated with cancer therapies [150, 151, 152]. Therefore, targeting allergic mediators may offer an opportunity to improve tolerability of therapies and enhance treatment response.

A more robust understanding of the TME components could help both stratify and guide treatment and shed light on non‐responder groups. For example, tumour infiltrating MC, eosinophils and secreted mediators have been proposed as contributors to underlying ICI resistance or treatment efficacy and the occurrence of irAE [52]. Eosinophil degranulation may create a tumour‐toxic environment that may be therapeutically targeted, for instance through inhibition of dipeptidyl peptidase IV (DPP4) [153, 154]. DPP4 cleaves the eosinophil chemoattractant CCL11, acting to decrease eosinophil recruitment; in mice, DPP4 inhibition increased CCL11 levels in tumours [154]. It is possible that therapeutic inhibition of DPP4 may enhance eosinophil recruitment to tumours and promote their anti‐tumour functions.

MC infiltration may be associated with worse outcomes, likely linked to histamine production and consistent with use of antihistamines during ICI treatment [155]. However, stimulation of MC with anti‐cancer IgE antibodies may harness their anti‐tumour potential. Separately, MC activation tests may complement the emerging use of the BAT to aid diagnosis and monitoring of allergic manifestations to anti‐cancer therapies [156]. Harnessing immune cells within the TME to drive anti‐cancer effects may therefore constitute a promising route for personalised medicine.

These insights exemplify how AllergoOncology can leverage insights from the study of allergic mechanisms to reprogram immune responses, reduce toxicities and boost cancer immunotherapy outcomes, fostering the development of precision medicine strategies to target both tumour resistance and mitigate toxicities.

## 8 Conclusion

**FIGURE 4:**
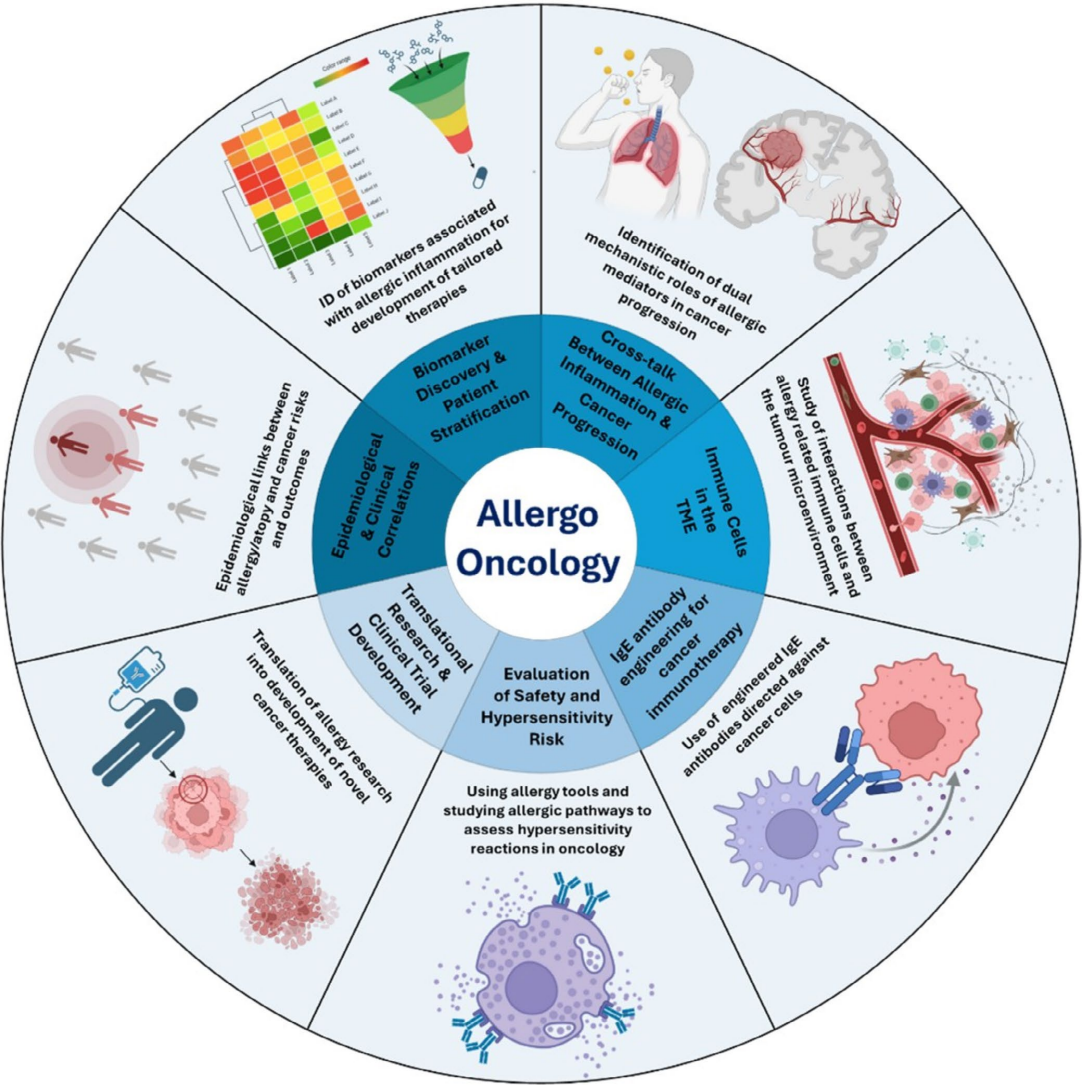
Key focus areas in AllergoOncology. The area of Epidemiological and clinical correlations studies the links between a history of allergy and the associated increased cancer risk and outcomes. Biomarker discovery and patient stratification focus on the identification of key biomarkers associated with disease that can enable the development of tailor‐made therapies specific for patients. Crosstalk between allergic inflammation and cancer progression aims to uncover the dual mechanistic roles of allergic mediators that may be protective against cancer or which may facilitate cancer progression. Studying immune cells in the tumour microenvironment (TME) explores how allergy‐related immune cells interact with the TME to influence both tumour growth and suppression. The area of IgE antibody engineering for cancer immunotherapy explores recombinant IgE‐based antibodies directed against cancer cells. Evaluation of safety and hypersensitivity risk investigates drug safety and tolerance, including assessing the risk and monitoring of type I hypersensitivity reactions using allergic tools which are translated to and established within oncology. Translational research and clinical trial development is centred around moving laboratory discoveries to the patient ‘bedside’ for clinical use to improve patient outcomes and prognosis. Created with BioRender.com.

Bridging the disciplines of allergy and cancer research, AllergoOncology is evolving across disciplines such as neuroscience, autoimmunity, dermatology and parasitology and expanding across the pipeline of immunotherapeutic discovery, from early‐stage to translational research, therapy innovation, clinical trials and reverse translation. AllergoOncology continues to integrate epidemiological analysis, the study and modulation of the TME, biomarker identification, tools to support patient safety and drug tolerability and the design of potentially life‐saving drugs. Cancer therapies can be driven by understanding allergic immunity, exemplified by the design and clinical translation of anti‐tumour IgE antibodies for cancer treatment, and by shedding light into the mechanisms by which IgE‐mediated anti‐tumour immunity can influence the TME and reverse tumour‐induced immune suppression. Insights from the study of allergic inflammation thus collectively inspire the development of innovative, personalised cancer therapies (Figure 4).

The AllergoOncology Working Group within the European Academy of Allergy & Clinical Immunology (EAACI) is at the forefront of these activities, promoting research into biomarker discovery and in‐depth investigation of how immune factors and cells such as granulocytes function in allergy and cancer. Several position papers in AllergoOncology offer unique perspectives into different aspects of the interphase between allergy and cancer (Table 1). These include exploring multiple allergic mechanisms underpinning cancer progression, exploiting allergy tools such as the BAT for oncology applications and developing novel approaches to harness Th2 immune responses and derive novel means of redirecting patient immunity against cancer. Insights from allergy can enhance how we understand cancer immunity, manage drug reactions and develop therapeutics.

At the interphase between allergy and cancer, AllergoOncology as a defined specialty bridges diverse disciplines in the complex and rapidly evolving landscape of translational and precision medicine, advancing knowledge and innovative therapeutic strategies to improve health and benefit many groups of patients.

## Author Contributions

Conceptualization: Alexandra J. McCraw, Sophia N. Karagiannis and Jakub Zydron. Methodology: Alexandra J. McCraw, Sophia N. Karagiannis, and Jakub Zydron. Investigation: Alexandra J. McCraw and Jakub Zydron. Writing – original draft preparation: Jakub Zydron, Alexandra J. McCraw, Sophia N. Karagiannis, Anishaa Balaji, Jitesh Chauhan, Jack Alder, Xinyi Chen, Anna M. F. Wiegman, and Aurelie Poli. Writing – review and editing: Alexandra J. McCraw, Sophia N. Karagiannis, Jakub Zydron, Daniel I. R. Spencer, Jitesh Chauhan, Joanna Jacków‐Malinowska, James McDonnell, and James Spicer. Supervision: Sophia N. Karagiannis, and Alexandra J. McCraw. Funding acquisition: Sophia N. Karagiannis, Daniel I. R. Spencer, James McDonnell, and James Spicer. All authors have read and agreed to the published version of the manuscript.

## Funding

The authors acknowledge support by Worldwide Cancer Research (24‐0087); UK Medical Research Council (MR/R015643/1), KCL member of the MRC Doctoral Training Partnership in Biomedical Sciences; Breast Cancer Now (147KL‐Q3); Cancer Research UK (CRUK City of London Centre Award) (C7893/A29290); Luxembourg National Research Fund (FNR) and Fondation Cancer Luxembourg (GRALL, C24/BM/18858278); Fund for Scientific Research ‐ FNRS (Télévie‐FNRS (RESTAGE 7.4576.23)). This research was supported by the King's Health Partners Centre for Translational Medicine. The views expressed are those of the author(s) and not necessarily those of King's Health Partners.

## Conflicts of Interest

S.N.K. and J.S. are academic founders and shareholders of Epsilogen Ltd. and have authored patents on antibody technologies for cancer. D.I.R.S. and J.C. are employed by Ludger Ltd. a company that commercialises glycan analytics. All other authors have declared that no conflicts of interest exist.

## Data Availability

The authors have nothing to report.
